# The value of organ and tissue biovigilance: a cross-sectional analysis

**DOI:** 10.3389/frtra.2024.1307946

**Published:** 2024-02-20

**Authors:** Aurora Navarro, Oscar Len, Eduardo Muñiz-Diaz, Joan-Lluis Vives Corrons, Beatriz Dominguez-Gil, Anna Vilarrodona, Jaume Tort

**Affiliations:** ^1^ Oganització Catalana de Trasplantaments (OCATT), Barcelona, Spain; ^2^ Associate teacher, University of Barcelona, Barcelona, Spain; ^3^ Infectious Diseases Department, Hospital Universitari Vall d'Hebron, Barcelona, Spain; ^4^ Department of Medicine, Universitat Autònoma de Barcelona (UAB), Barcelona, Spain; ^5^ Banc de Sang i Teixits, Barcelona, Spain; ^6^ Professor emeritus, Hospital Clínic de Barcelona, Barcelona, Spain; ^7^ General Direction, Organización Nacional de Trasplantes, Madrid, Spain; ^8^ Barcelona Tissue Bank, Banc de Sang i Teixits, Barcelona, Spain; ^9^ Vall d'Hebron Institute of Research (VHIR), Barcelona, Spain

**Keywords:** biovigilance system, transplantation, serious adverse events, serious adverse reactions, quality and safety, retrospective analysis, tissue–organ cross-sectional analysis

## Abstract

**Introduction:**

Biovigilance (BV) systems aim to improve the quality and safety of tissues and organs for transplantation. This study describes the Catalan BV system and analyzes its utility.

**Methods:**

It is a retrospective analysis of notifications on serious adverse events (SAEs) and reactions (SARs) since the implementation of the BV system (2008 for tissues and 2016 for organs) until 2020. Variables are presented to describe the most common critical steps of the pathway and complications associated with the quality and safety of tissues and organs.

**Results:**

A total of 154 and 125 notifications were reported to the Tissue and the Organ BV systems, respectively. Most SAEs were related to unexpected donor diseases and implemented actions were assured on those deemed preventable. Regarding SARs, donor-transmitted infections and malignancies (only organs) were the most common, followed by graft failure (tissues) and process-related (organs). The incidence of SAEs and SARs related to tissue was 3.44‰ and 0.22‰, respectively. The corresponding figures for organs were 31.48‰ and 8.8‰, respectively.

**Discussion:**

The analysis of the notifications to the Catalan BV systems has provided useful information about existing risks associated with the quality and safety of tissues and organs, and enabled the implementation of actions targeted to diminish risks and mitigate damage.

## 1 Introduction

The transplantation of human tissues and organs has a profound impact upon the survival and quality of life of patients, by restoring essential functions where no comparable effective alternative exists (1). However, donation and transplantation are complex processes, with clinical, organizational, ethical, sociocultural, and religious implications, which limit patients' access to transplant therapies (2, 3).

Despite strict donor selection criteria, comprehensive pre-donation and pre-allocation to recipient testing (4, 5), and controlled preservation of tissues and organs to guarantee their integrity, there are risks associated with the different steps of the pathways that make tissues and organs available to patients (6). Moreover, tissues and organs of human origin are not exempt from residual risks, which may lead to unexpected complications, such as the transmission of infections, malignancies, or other diseases (7–9).

**Figure 1:**
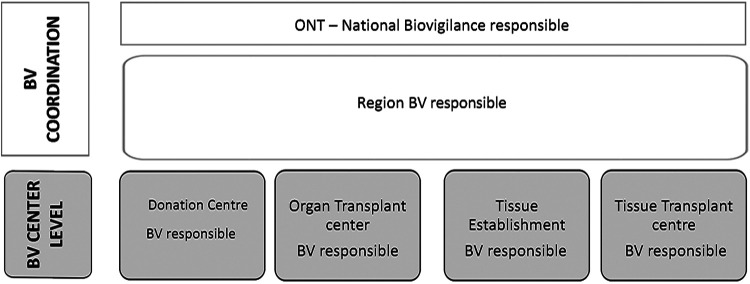
Spanish biovigilance structure.

Biovigilance (BV) systems analyze any unexpected occurrence related to the obtaining or the quality and safety of tissues and organs from living or deceased donors, communicating and managing them to minimize damage (10). BV programs have been implemented in a diverse, uneven manner worldwide (10–14). The Additional Protocol to the Council of Europe Convention on Human Rights and Biomedicine (15) and the World Health Organization (WHO) Guiding Principles on Human Cell, Tissue and Organ Transplantation (16) establish the need to incorporate BV and surveillance systems into transplantation programs. The establishment of BV has become a mandatory standard in the European Union after Directives 2004/23/EC (17) and 2010/53/EU (18) entered into force. In Spain, the implementation of both Directives led to the creation of two BV systems, one National Tissue BV System in 2008 (through Royal Decree 1301/2006) (19) and one National Organ BV System in 2016 (through Royal Decree 1723/2012) (20). Both systems were set up in alignment with the structure of the tissue and organ Spanish Network, based on three levels of organization (Figure 1): the Spanish National Transplant Organization [Organización Nacional de Trasplantes (ONT)] responsible for the overall management and coordination; 17 regional coordination units; and, at the center level, transplant coordinators, transplant teams, and tissue establishments (TEs).

The regional coordination units are responsible for the management and coordination of BV in the corresponding region. The BV System in Catalonia (Spanish region of 7.7 million inhabitants) is set up upon 22 donation centers, 3 TEs, 8 transplant hospitals with 20 organ transplant programs, the 24/7 organ exchange coordination unit and more than 200 tissue transplant centers. The Catalan BV system incorporates a medical BV officer who coordinates the BV system and one multidisciplinary advisory committee for the investigation of serious adverse events (SAEs) and reactions (SARs), the impact analysis, and action proposals (Supplementary Tables S2, S5).

The main objective of this study was to analyze the utility of BV systems by describing tissue and organ BV data in the region of Catalonia. In particular, we aimed at summarizing the types of SAEs and SARs reported in the tissue and organ field, identifying critical points in the pathway, as well as analyzing the utility of the actions implemented with the purpose of enhancing the protection of living donors and recipients.

## 2 Materials and methods

### 2.1 Study design and data sources

A retrospective analysis was performed of all SAEs and SARs reported related to tissues (13 years) and organs (5 years) since the implementation of the BV system (2008 for tissues, 2016 for organs) until 2020.

The definitions of SAE and SAR were those described in Directives 2004/23/EC (17) and 2010/53/EU (18). In brief, an SAE (risk of serious harm) is any untoward occurrence associated with the procurement, testing, processing, storage, and distribution of tissues or organs that might lead to a serious complication in the living donor or the recipient. An SAR (serious harm) refers to an unintended response in the living donor or the recipient that might be associated with any stage of the donation–transplantation chain, or to the quality and safety of tissues or organs, and is considered a serious complication. A serious complication consists of the transmission of communicable diseases, death or life-threatening, disabling or incapacitating conditions, or which might result or prolong hospitalization or morbidity.

**Figure 2:**
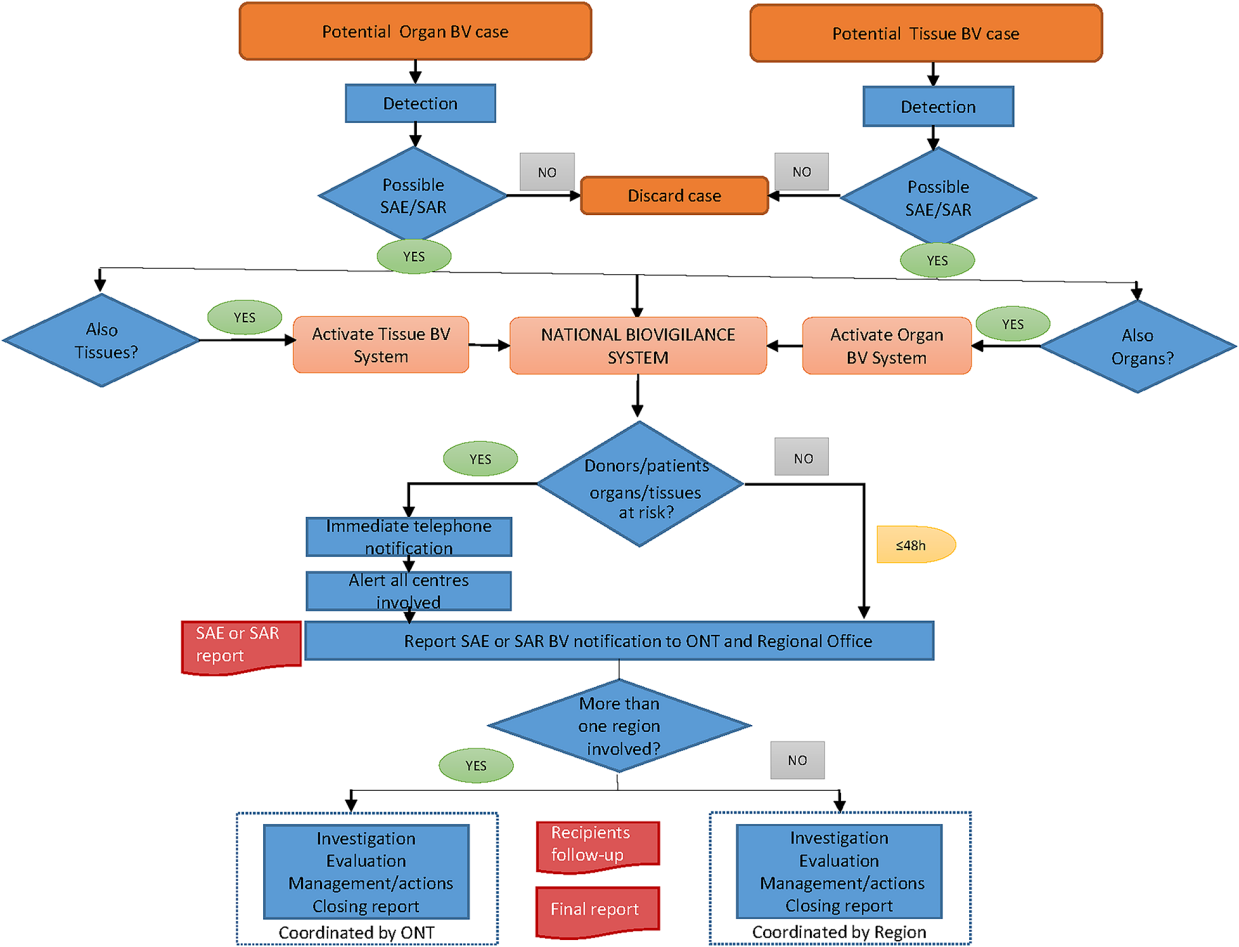
Spanish biovigilance system organization.

Data were obtained from the Catalan BV Registry (rBioVc) (21), when a BV notification only involved Catalan centers, and from the ONT BV registry (Figure 2) when centers from other regions were affected. Other sources of information were the Donation and Transplantation Registry of Catalonia (rDTx) and the patients' electronic health records (HC3) of the Catalan Health Service.

The study protocol was approved by the Ethics Committee of the Vall d'Hebron University Hospital [reference PR(BST) 323/2016)]. All data were processed according to Regulation (EU) 2016/679 of the European Parliament and of the Council (22), the provisions of the Spanish Organic Law 3/2018 (23), and other applicable regulations on data protection.

### 2.2 Variables

**Figure 3:**
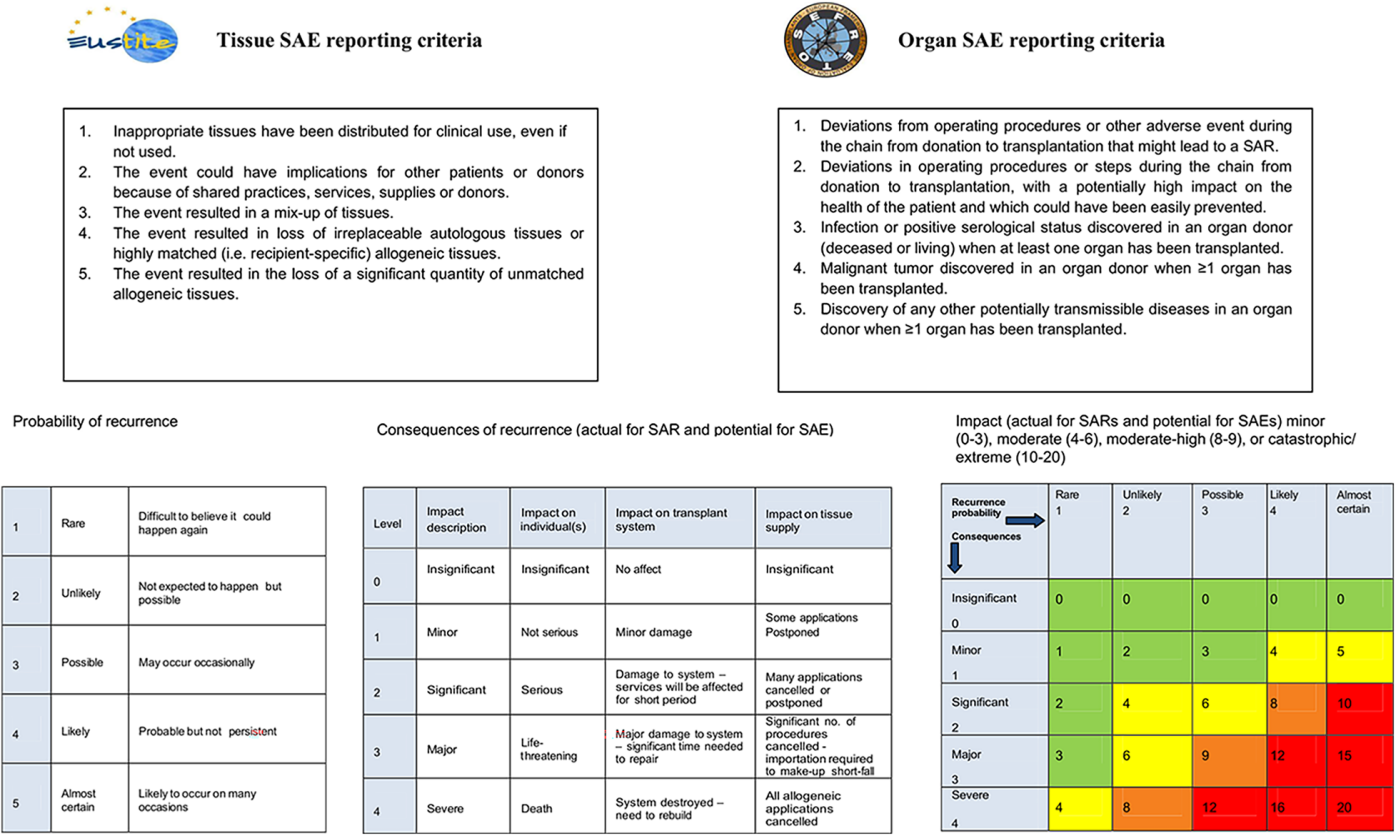
Tissue/organ SAE reporting criteria and Eustite impact tools.

Variables collected for SAEs included the reporting criteria (Figure 3), from the Eustite Project (24) for tissues and the Efretos project (25) for organs. Other variables included were as follows: the reporter; the risk; the stage; the cause; the number of centers involved (for organs); and the actions proposed by the BV advisory committee after investigation.

**Figure 4:**
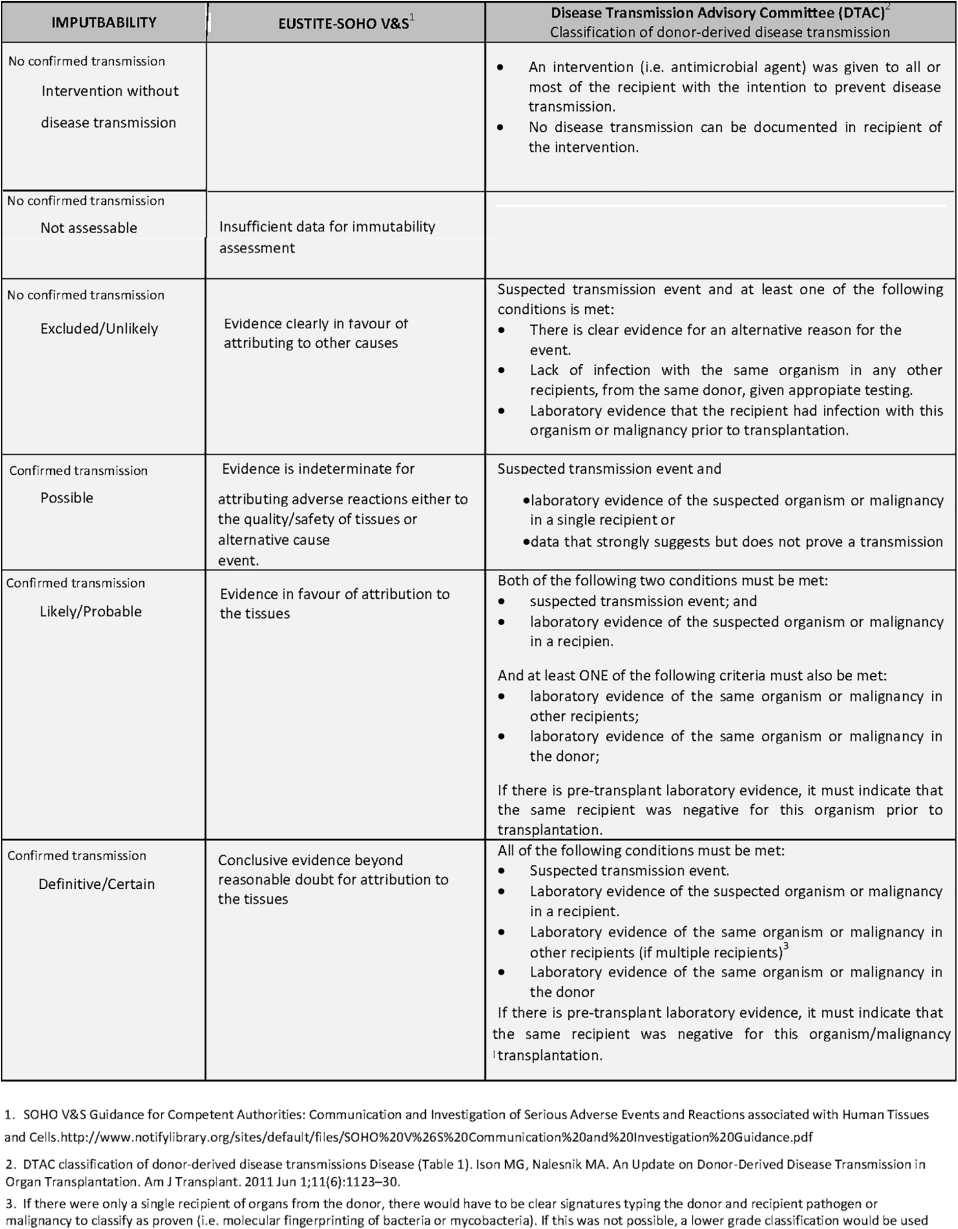
Tissue (Eustite-SoHO) and organ (DTAC-notify) imputability criteria.

SARs were described according to the organ or tissue recipient with SAR, the type of SAR (as per the Notifylibrary taxonomy (26, 27)), detection time, and imputability [based on the Eustite project for tissues (24) and the Disease Transmission Advisory Committee (DTAC) (28) for organs] (Figure 4). In process-related issues, imputability was classified as “certain-process related”.

In order to prioritize actions once cases were closed, harmful impact was calculated (actual for SAR or potential for SAE) by multiplying the likelihood of recurrence and the highest score of consequence (for the individual, the system, or the distribution of tissues and organs). These tools are those proposed by the Vigilance and Surveillance of Substances of Human Origin (SoHO V&S) (29) project and the Eustite project (24). The impact matrix classification table applied in this study has four levels: minor (0–3); moderate (4–6); moderate/high (8–9); or catastrophic/extreme (10–20). Finally, the preventability capacity (considered when changing or implementing a test or procedure could have avoided the notification) was considered.

### 2.3 Analysis

Qualitative variables were described as frequencies, percentages and per thousand. The incidence of SAEs was calculated by dividing the number of SAEs recorded during the study period by the number of donations registered within the same time frame. In contrast, the incidence of SARs was calculated as the number of patients with certain, probable, and possible (CPP) SARs notified during the study period, divided by the number of transplants registered within the same time frame. The potential for disease transmission was calculated by dividing the number of recipients with a proven, probable, or possible SAR by the total number of recipients at risk. In addition, the mortality rate was calculated as the number of deaths divided by the total number of transplants.

Qualitative variables were compared using the chi-square test, and the significance threshold was set at a two-sided alpha of 0.05. Statistical analyses were performed using STATA 17 and Microsoft Excel.

## 3 Results

### 3.1 Donations, transplants, and notifications during the study period

Between 2008 and 2020, 34,306 tissues donors and 149,891 tissues were distributed for clinical use. Between 2016 and 2020, 2,287 organ donors were registered and 5,569 organ transplants were performed (Supplementary Table S1). During the corresponding period, 154 and 125 BV notifications related to tissues and organs were made, respectively (an average of 12 notifications per year for tissues and 25 for organs). Regarding tissues, 118 (77%) notifications were classified as SAEs and 36 (23%) as SARs. With respect to organs, 72 (58%) notifications were SAEs and 53 (42%) were SARs. The imputability of SARs was deemed CPP in 35 tissue recipients and 49 organ recipients.

### 3.2 SAEs in the tissue BV system

Of the 118 SAEs, more than half were reported by the TE and the most common criterion for reporting (73.7%) was an unexpected result from donor testing or tissue culture that compromised the quality or the safety of the tissue after it had been distributed (mainly corneas due to their expiry date). In contrast, system, equipment, and material failures and human errors accounted for only 13% of SAEs (Table 1).

**Table 1:** Characteristics of serious adverse event reports related to tissue donation *N (%): N* = 118.

Reporter	
Tissue establishment	64 (54.2)
Transplant center	34 (28.8)
Donor center	20 (17.0)
Criteria for reporting serious adverse events	
Inappropriate tissues/cells distributed for clinical use^a^	87 (73.7)
The event could have possible implications for other patients or donors	22 (18.6)
The event resulted in a mix-up of tissues/cells	3 (2.5)
The event resulted in the loss of irreplaceable autologous tissues/cells or highly matched allogeneic tissues/cells	3 (2.5)
The event resulted in the loss of a significant quantity of unmatched allogeneic tissues/cells	3 (2.5)
Risk origin	
Donor	65 (55.1)
Tissue	53 (44.9)
Stage of occurrence	
Donor selection	5 (4.2)
Donor testing	66 (55.9)
Procurement cultures	7 (5.9)
Processing cultures	5 (4.2)
Storage	1 (0.8)
Tissue selection	0 (0.0)
Tissue allocation	5 (4.2)
Tissue distribution	1 (0.8)
Tissue transport	2 (1.7)
Culture of implanted tissue at transplant center	22 (18.6)
SARs in recipients of organs obtained from the same donor	4 (3.4)
Cause	
Donor disease	60 (50.8)
Tissue quality	42 (35.6)
System failure	9 (7.6)
Human error	3 (2.5)
Materials failure	2 (1.7)
Equipment failure	1 (0.9)
Others	1 (0.9)

^a^ Donor or tissue culture results compromise tissue quality and/or safety after tissue distribution (mainly corneas due to their expiry date).

**Figure 5:**
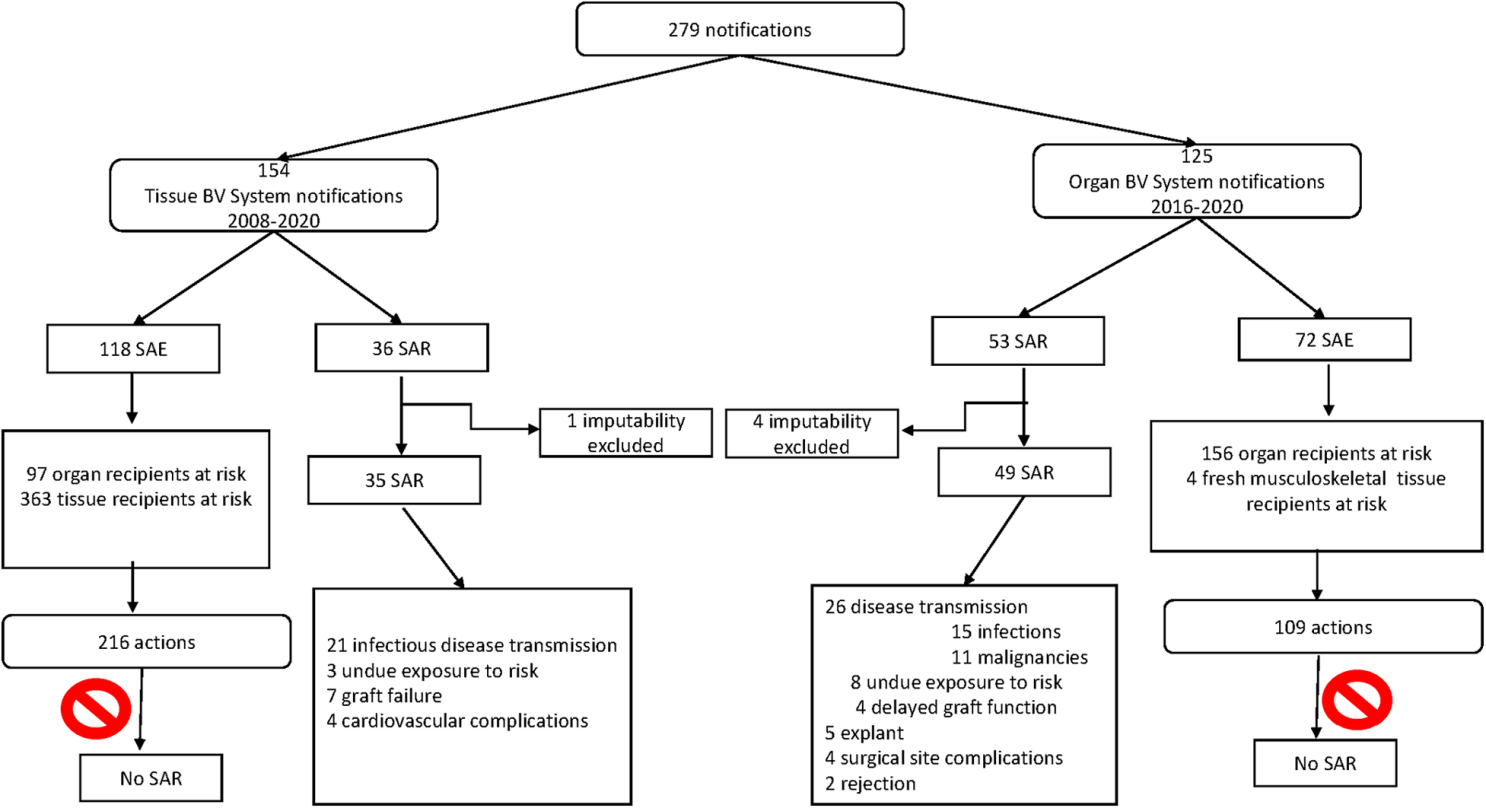
Catalan biovigilance data for tissues (2008–2020) and organs (2016–2020).

The potential impact of SAEs was mostly moderate (97%) and only two had an impact on tissue supply (28 corneas were discarded due to a disqualified incubator and 40 processed bone donors immobilized under the suspicion of a contaminated processing solution). The potential individual impact was upon 363 tissue recipients and 97 organ recipients (Figure 5). A total of 216 actions were implemented and 31 (26%) SAEs were considered preventable exploring the following actions: (1) change from Rapid Plasma Reagin test to *Treponema pallidum* hemagglutination assay (TPHA) before donation (2021); (2) screen all tissue donors using hepatitis B virus nucleic acid testing (HBV NAT); (3) incorporate a checklist of standard operating procedures (SOPs) for distribution; (4) include final validation of a tissue before distribution; and (5) intensify personnel training to prevent human errors (Table 2 and Supplementary Table S2).

**Table 2:** Actions implemented after serious adverse events related to tissue donation, *N (%): N* = 216.

Learning	5 (2.3)
No prophylaxis for avascular tissues (cornea recipient)	4 (1.8)
Return of corneal tissue to tissue establishment for quality control	1 (0.5)
Improvement	22 (10.2)
Validation of serological tests	3 (1.4)
Tissue validation	6 (2.8)
Change and/or review of procedures	11 (5.1)
Discard of equipment (incubator)	1 (0.45)
Implementation of nucleic acid testing (NAT)	1 (0.45)
Prevention	153 (70.8)
Discard of tissues	58 (26.8)
Follow-up of organ recipients	47 (21.8)
Follow-up of tissue recipients	48 (22.2)
Correction	36 (16.7)
Prophylactic treatment of tissue recipients	24 (11.1)
Prophylactic treatment of organ recipients	12 (5.6)

### 3.3 SARs in the tissue BV system

Of 36 SARs reported, 83% involved recipients of ocular and musculoskeletal tissue and more than half (61.1%) consisted of suspected donor-transmitted infections (Table 3). For ocular infections, fungal infection was predominant before day 90. Among recipients of musculoskeletal tissues, bacterial infections before day 30 were the most frequent (Supplementary Table S3). The second type of SAR in frequency was graft failure, occurring up to day 150 after cornea transplantation and within the first 40 days after the transplantation of cardiovascular tissue. There was one late tendon graft failure occurring on day 300.

**Table 3:** Characteristics of serious adverse reaction reports related to tissue transplantation according to the recipients involved, *N (%): N* = 36.

Tissues involved	
Ocular	20 (55.6)
Musculoskeletal	10 (27.8)
Heart valves	2 (5.6)
Arteries	2 (5.6)
Autologous serum (eye drops)	2 (5.6)
Cause	
**Disease transmission**	**22 (61.1)**
Infection	22 (61.1)
Bacterial	7 (19.4)
Fungal	12 (33.3)
Not determined	3 (8.3)
Malignancy	0 (0.0)
**Undue exposure to risk/intervention**	**3 (8.3)**
Transplant cancelled	1 (2.8)
Error in product selection	2 (5.6)
**Other**	**11 (30.6)**
Graft failure	7 (19.4)
Cardiovascular reaction	4 (11.2)

Imputability was considered unlikely only in one case (evidence clearly showed that the patient had a predisposing condition). Among the the 35 SAR with CPP imputability, 92% deemed to have a moderate impact. Only in three recipients was the impact considered moderate/high, corresponding to two recipients of cardiovascular tissue and one recipient of a corneal transplant. Only three non-transmissible SARs (8%) modified SOPs on final cornea quality validation and preservation and packaging verification (Supplementary Table S3).

### 3.4 SAEs in the organ BV system

From a total of 72 SAEs, 44% were reported by the transplant center (Table 4) followed by the TE (30%). A malignancy identified in the donor at the pathology/autopsy examination after solid organ transplantation had taken place was the most frequent reporting criteria (40%), followed by donor blood testing (28%) due to positive HBV NAT, positive syphilis serology, and parasites screening (results after donation). Therefore, the main cause for opening a notification was the identification of a disease in the donor unknown before transplantation (67%), while system or equipment failure and human errors accounted for 33% of cases. Supplementary Table S4 summarizes the frequency of SAEs according to donor type, which was statistically significantly lower in living vs. deceased donation (*p* < 0.0001).

**Table 4:** Characteristics of serious adverse event reports related to organ donation, *N (%): N* = 72.

Reporter	
**Transplant center**	**32 (44.4)**
Catalan transplant center^b^	26 (36.1)
Other regions transplant center^c^	6 (8.3)
**Donor center**	**17 (23.6)**
Catalan donation center^b^	12 (16.6)
Other regions donation center^c^	5 (7)
**Tissue establishment**	**21 (29.2)**
Catalan TE^b^	20 (27.8)
Other region TE^c^	1 (1.4)
**Immunology laboratory**	**2 (2.8)**
Number of hospitals/tissue establishments involved in each SAE	
1	3 (4.2)
2	28 (38.9)
3	25 (34.7)
4	11 (15.3)
5	4 (5.6)
6	1 (1.4)
Reporting criteria^a^	
Deviations from operating procedures or other adverse event during the chain from donation to transplantation that might lead to a SAR	8 (11.1)
Deviations in operating procedures or steps during the chain from donation to transplantation, with a potentially high impact on the health of the patient and easily prevented	15 (20.8)
Infection or positive serological status discovered in an organ donor (deceased or living) when at least one organ has been transplanted	20 (27.8)
Malignant tumor discovered in an organ donor when ≥1 organ has been transplanted	29 (40.3)
Risk origin	
Donor	54 (75.0)
Organ	18 (25.0)
Stage of occurrence	
Donor characterization	8 (11.1)
Blood testing	19 (26.4)
Cultures (bronchial aspirate/blood)	5 (7.0)
Procurement	9 (12.5)
Perfusion/preservation/packaging	6 (8.3)
Transport	0 (0.0)
Preservation solution	1 (1.4)
Pathology analysis	18 (25.0)
Autopsy	5 (7.0)
Follow-up (living donors)	1 (1.4)
Cause	
Donor disease without transmission	40 (55.6)
System failure	13 (18.0)
Organ quality	8 (11.1)
Human error	9 (12.5)
Equipment failure	2 (2.8)

SAE, serious adverse event; SAR, serious adverse reaction; TE, tissue establishment.

^a^ Reporting criteria from European Framework for the Evaluation of Organ Transplants (Efretos). Final Deliverables 2011.

^b^ OCATT BV registry.

^c^ ONT BV registry.

The potential impact of SAEs was mostly moderate (92%) upon 156 organ and 4 musculoskeletal tissue recipients (Figure 5); however, 20 corneas were considered suitable despite malignancy, because malignancy does not contraindicate cornea transplantation. There were only 2 (3%) moderate/high impact cases due to the identification of an active malignancy in the donor (enteral adenocarcinoma and thyroid cancer) (Supplementary Table S5). None of the SAEs had any impact on the donation system or on organ supply.

A total of 109 actions were implemented while 20 (28%) SAEs were deemed preventable executing the following actions: (1) results of *T. pallidum* antibodies available before transplantation; (2) requirements for blood testing, including automatic transcription (implemented in 2022); (3) elaboration of algorithms for decision-making when new techniques are incorporated in the serology or HLA lab (2019); (4) training staff for a correct donor characterization; (5) review, change, and incorporation of a checklist of SOPs for blood sampling, packaging, and preservation; and (6) creation of an urgent alert from the pathology department to the BV system when results compromise the recipient’s safety (Table 5).

**Table 5:** Actions implemented for serious adverse events related to organ donation, *N (%)*: *N* = 109.

Learning	4 (3.7)
Include post-transplant parasite screening (*Strongyloides stercoralis, Schistosoma,* and *Plasmodium falciparum* if donor was born in or has travelled to an endemic area^a^	4 (3.7)
Improvement	23 (21.1)
Review procedures and staff training	8 (7.3)
Repeat and validate blood testing	4 (3.7)
Review of procurement techniques	4 (3.7)
Review of procedures for organ preservation and packaging	5 (4.5)
Pathology alert	2 (1.9)
Prevention	59 (54.1)
Discard of tissues	31 (29.4)
Follow-up of organ recipients	20 (18.3)
Follow-up of tissue recipients	1 (0.9)
Review vaccination	2 (1.8)
Monitoring recipient serology/NAT	5 (4.5)
Correction	23 (21.1)
Prophylactic treatment of organ recipients	16 (14.7)
Prophylactic treatment of tissue recipients	2 (1.8)
Artery repair	4 (3.6)
Liver explant	1 (1)

SOP, standard operating procedures.

^a^ *Toxoplasmosis* (Spain endemic area) and *Trypanosoma cruzi* (immigration and traveling) are routine organ donor tests.

Regarding donor malignancies (seven were classified as of unacceptable risk according to the Council of Europe Guide (30)), no transmission occurred after a follow-up of 2–5 years. However, for the mesenteric adenocarcinoma SAE, a liver-kidney transplant was explanted 3 weeks after transplantation. None of the recipients developed a SAR.

### 3.5 SARs in the organ BV system

From 53 SARs reported, 83% consisted of complications developed by kidney and liver recipients, and more than half were donor-transmitted infections or malignancies, unknown at the time of donation (Table 6), with 43% non-transmissible SARs (undue exposure to risk, explant, delayed graft function, surgical site complications, and detrimental immunization, mainly classified as process-related). Detection time (Supplementary Table S6) for bacterial infection was less than 14 days, 30–850 days for viral infections, 5–120 days for parasites, and less than 1 week for fungal infections, except for a case of histoplasmosis (day 1700). The malignancy detection time varied depending on the type of cancer, from kidney and urinary tract and blood and lymphoid (46–90 days), lung and lower respiratory system (1 year), gastrointestinal (2 years), and a case of cholangiocarcinoma (4 years), which was deemed donor-derived rather than donor-transmitted.

**Table 6:** Characteristics of serious adverse reaction reports related to organ transplantation according to the organ recipients involved, *N (%): N* = 53.

Organs involved	
Kidney	33 (62.3)
Liver	11 (20.8)
Lung	5 (9.4)
Heart	3 (5.7)
Pancreas and kidney	1 (1.9)
Cause	
Disease transmission	30 (56.6)
Infection	17 (32.1)
Bacterial	5 (9.4)
Viral	4 (7.6)
Fungal	4 (7.6)
Parasitic	4 (7.6)
Malignancy	13 (24.5)
Undue risk exposure	8 (15.1)
Transplant cancelled^a^	7 (13.2)
Inappropriate clinical application	1 (1.9)
Miscellaneous complications	13 (24.6)
Delayed organ function	4 (7.6)
Explant	5 (9.4)
Surgical accident	4 (7.6)
Immunological complications: detrimental immunization	2 (3.7)

^a^ Transplants cancelled due to inappropriate organ perfusion (*n* = 2), cysts (*n* = 1), and vascular abnormalities (*n* = 3) and a liver mass classified as cholangiocarcinoma (*n* = 1) but 24 h after discarding the liver, a biliary adenoma was confirmed.

Imputability was excluded in 4(8%) SARs (two infections and two malignancies) and among the remaining 49 CPP SARs, 36 (74%) were certain (including 23 process-related cases) showing an unexpected infectious transmissions of 0.23% and malignancies 0.16%. Taking into account imputability (Supplementary Table S6), 13 donors transmitted an infection to 15 out of 40 (38%) recipients at risk, which became fatal in 3 (20%) cases and 9 donors transmitted a malignancy to 11 out of 26 (42%) recipients at risk, with a fatal outcome in 6 (55%) cases. In fact, among infectious disease transmissions, only one donor transmitted parasites to more than one recipient, and among malignancies, only soft tissue/sarcoma and gastrointestinal malignancy were transmitted (Supplementary Table S1).

In terms of the impact of SARs, 78% were deemed to have moderate impact, 8 (16%) moderate/high impact, and 2 (4%) extreme impact (Supplementary Table S6); two kidneys were explanted 1 month after transplantation due to a donor autopsy revealing a lung malignancy. Both recipients were re-transplanted 3 and 4 years after explant and none had malignancy transmission after 7 years of close monitoring. Significant actions due to non-transmissible SARs included a double verification to include recipients on the waiting list, review of HLA SOPs and staff training, inclusion of the ABO blood group in the verification checklist before the transplant, ensuring and confirming the laterality terminology, and promoting continuous training on SOPs for organ procurement and packaging.

### 3.6 Incidence of SAEs and SARs during the study period

During the study period, the incidence of SAEs and CPP SARs related to tissue donations and transplants was 3.44‰ and 0.22‰, respectively, while the incidence of SAEs and SARs related to organ donations and transplants was 31.48‰ and 8.8‰, respectively.

## 4 Discussion

The present study is a cross-sectional analysis of 279 notifications from the tissue and organ BV systems in Catalonia. It reveals that risks detected (SAE) are more common than unexpected complications (SAR) in both systems. SAEs most commonly consist of unexpected positive results in donor blood testing and tissue cultures in the tissue field, and of infectious diseases and malignancies identified in the donor in the organ setting. Approximately 27% of SAEs are preventable in both systems (mainly for system and equipment failure and human errors). Thus, actions were assured for implementing changes in SOPs, incorporating checklists, and training staff as the main tools to knock down these events. Ensuring results from *Treponema pallidum* antibodies are available prior to transplantation and universal screening of tissue donors by HBV NAT before distribution have cleared away specific risks. None of the 190 EAGs investigated and the 325 actions proposed by the BV advisory committee have resulted in a SAR among the 620 at-risk recipients, demonstrating the importance of notification and its management.

With respect to SARs, these were predominantly cases of donor-transmitted infections for tissues as well as malignancies for organs, affecting mostly a single recipient from the same donor. The study showed one SAR per approximately 1,000 transplants of cardiovascular and cornea tissue, with a much lower incidence in bone transplantation and none for the transplantation of skin and amniotic membrane. The incidence in the organ setting was of one SAR per 100 organ transplants (highest for the liver, at 1/89 transplants, and lowest for the kidney, with1/127 transplants).

The organ BV System has higher notification incidence (9 folds for SAE and 41 for SAR) and a more harmful impact compared to the one observed in the tissue BV System. This might be attributed to many factors, such as the higher risks assumed in organ transplantation due to organ shortage (31), time constraints for performing tests and cultures without the possibility of sterilization, issues related to organ preservation (32), use of immunosuppressive therapies in recipients, and the more structured and centralized clinical outcome registries that exist in the organ vs. the tissue setting, which facilitates the detection of SARs.

To the best of our knowledge, this is the first study to detail the risks identified in organ and tissue donation programs permitting the linking of organs and tissues independently of the BV system initiated and compiling the actions adopted to reduce risks and mitigate damage (11, 33). In fact, some unique organizational aspects, such as active communication and training among national and regional medical BV offices, donation and transplant network structures, and the BV advisory committee, have contributed to build a well-structured model of BV. It is also important to emphasize the role of TEs, which reported more than half of SAEs for the tissue BV system and almost 30% for the organ BV system.

Regarding tissue data, the percentage of SAEs vs. SARs reported in our study is comparable to European data, with SAEs accounting for more than 75% of the notifications (11). The incidence of SARs in the tissue setting (0.022%) is also similar to that reported for non-reproductive cells and tissues distributed in the European Union (range 0.013%–0.28%). However, there are differences in the causes of SAEs. In our study, most SAEs in the tissue field consisted of the identification of transmissible diseases in the donor or positive tissue cultures. In contrast, SAEs usually consisted of system failures (in France) (33) and human errors (in the European Union) (34). In terms of SARs, graft failure was the leading cause in the European Union, while in our study, the leading cause was infectious disease transmission. With respect to the percentage of SARs by type of tissue, 28% of SARs in our study and the European Commission involved musculoskeletal tissue, despite accounting for 78% of tissue transplants in our study and 58% in the European report. Conversely, ocular tissue accounted for 18% and 28% of tissue transplants but was involved in 55% and 46% of SARs in our analysis and the European report, respectively (34). These differences may be attributed to less significant processing methods available for corneal tissue (without the possibility of sterilizing) and a shorter interval time between donation and transplantation, with a maximum of 21 days for hypothermic corneal tissue (35) and 7 weeks for organ culture (36). In the latter case, the intrinsic peculiarity is that cornea distribution is performed with culture results pending (37), with the possibility of positive results once corneas have already been transplanted but having the advantage that, once the risk is known, damage can be prevented (as shown in this study).

Concerning organ data, the higher percentage of SAEs compared to SARs is similar to other reports (38, 39). The leading cause of SAEs in our study was donor disease without transmission, which is similar to the United Kingdom (40) but differs from other studies describing transcription, procurement, or transport errors as the predominant cause (33, 39, 41). However, our data are similar to the findings reported by Kaul et al. (42) with regard to potential disease transmission from the donor to exposed recipients (38% for infections and 42% for malignancies in our study vs. 46% and 57%, respectively) and mortality (20% vs. 15% for infections and 55% vs. 38% for malignancies). Remarkably, the percentage and type of microorganisms transmitted are similar: one in three infections were caused by bacteria [mainly *Escherichia coli* (43), *Klebsiella* spp. (44), and *Pseudomonas* spp. (45)]; one in four infections were due to virus (46); approximately one in five were fungal [mainly *Candida* spp. (47)], and one in seven were caused by parasites, primarily *Strongyloides stercoralis* (48, 49).

This study has some limitations. First, even though reporting is mandatory by law, the wide range of professionals involved, the lack of previous experience in reporting to understand the benefits of culture in safety, the difficulties in understanding which events or reactions must be notified and managed by the BV system, and the failure to provide an online reporting system likely result in an under-reporting of cases. Nevertheless, data from the Catalan BV system shows an increasing trend in the reporting of SAEs and SARs, as in the Spanish data (50). A major limitation when analyzing the data presented is the difficulty in comparing them with other organ and tissue BV systems due to the small number of published reports, the lack of evolution of BV results associated with the number of donations and transplantations (which hinders the comparison of incidences), and the variety of reporting and imputability criteria.

Future improvements for the Catalan BV system should be focused on establishing an online reporting system and active dissemination of BV results, increasing patients' follow-up from 2 to 5 years to cover unexpected malignancies transmission, enable continuous education, and introduce a BV auditing program.

In summary, our study provides a detailed description of the SAEs and SARs notified to a regional biovigilance office over the years and demonstrates the utility of their subsequent management providing reliable data to support more accurate risk management decisions and further guidance.

## Data Availability

The original contributions presented in the study are included in the article/**Supplementary Material**, further inquiries can be directed to the corresponding author.

## References

[1] Grinyó JM. Why is organ transplantation clinically important? Cold Spring Harb Perspect Med. (2013) 3(6):1–10. 10.1101/cshperspect.a014985 PMC3662355 23732857

[2] Cotrau P Hodosan V Vladu A Daina C Daina LG Pantis C. Ethical, socio-cultural and religious issues in organ donation. Maedica. (2019) 14(1):12–4. 10.26574/maedica.2019.14.1.12 31123506 PMC6511665

[3] Robson NZMH Razack AH Dublin N. Review paper: organ transplants: ethical, social, and religious issues in a multicultural society. Asia Pacific J Public Heal. (2010) 22(3):271–8. 10.1177/1010539509357446 20460294

[4] Domínguez-Gil B Delmonico FL Shaheen FAM Matesanz R O’Connor K Minina M The critical pathway for deceased donation: re-portable uniformity in the approach to deceased donation. Transpl Int. (2011) 24(4):373–8. 10.1111/j.1432-2277.2011.01243 21392129

[5] Sandiumenge A Domínguez-Gil B Pont T Sanchez Ibanez J Chandrasekar A Bokhorst A Critical pathway for deceased tissue donation: a novel adaptative European systematic approach. Transpl Int. (2021) 34(5):865–71. 10.1111/tri.13841 33559299 PMC8251811

[6] Rosales B Hedley J De La Mata N Vajdic CM Kelly P Wyburn K Safety and biovigilance in organ donation (SAFEBOD): protocol for a population-based cohort study. JMIR Res Protoc. (2020) 9(10):e18282. 10.2196/18282 33104005 PMC7652689

[7] López-Menchero C Alvarez M Fernández P Guzmán M Ortiz-de-Salazar MI Arbona C. Evolution of the residual risk of HBV, HCV and HIV transmission through blood transfusion in the region of Valencia, Spain, during a 15-year period (2003–2017). Blood Transfus. (2019) 17(6):418–427. 10.2450/2019.0058-19 31403928 PMC6917534

[8] White SL Rawlinson W Boan P Sheppeard V Wong G Waller K Infectious disease transmission in solid organ transplantation: donor evaluation, recipient risk, and outcomes of transmission. Transplant Direct. (2018) 5(1):e416. 10.1097/TXD.000000000000085210 30656214 PMC6324914

[9] Eccher A Girolami I Motter JD Marletta S Gambaro G Momo REN Donor-transmitted cancer in kidney transplant recipients: a systematic review. J Nephrol. (2020) 33(6):1321–32. 10.1007/s40620-020-00775-4 32535833 PMC7701067

[10] Singh S Chandel S Sarma P Reddy DH Mishra A Kumar S Biovigilance: a global perspective. Perspect Clin Res. (2019) 10(4):155–62. 10.4103/picr.PICR_89_18 31649864 PMC6801993

[11] Schuantes-Paim SM Wachholz LF Knihs NDS De Oliveira PC Roza BA Schirmer J. Adverse events reporting systems in cells, organs, and tissues donation and transplantation: scoping review. Transplant Proc. (2023) 55(6):1352–58. 10.1016/j.transproceed.2023.04.032 37246129

[12] Mehakovic E. Safety and vigilance in organ donation for transplantation. Transplantation. (2017) 101:S97. 10.1097/01.tp.0000525129.87363.30

[13] Schuantes-Paim SM Roza BA Knihs NDS Time elapsed between cells, tissues, and organs donation and transplantation and adverse events detection. Transplant Proc. (2023) 55(6):1359–61. 10.1016/j.transproceed.2023.03.039 37105826

[14] Martinière K Lucas S Zorzi P. Events and adverse reactions in biovigilance: descriptive analysis of French national data following a four-year practical experience. Transfus Clin Biol. (2008) 15(4):179–89. 10.1016/j.tracli.2008.04.008 18538607

[15] Council of Europe. Additional Protocol to the Convention on Human Rights and Biomedicine concerning Transplantation of Organs and Tissues of Human Origin. European Treaty Series—No. 186. (2002). Available at: https://rm.coe.int/1680081562

[16] Sixty-Third World Health Assembly WHO. WHO guiding principles on human cell, tissue and organ transplantation. Cell Tissue Bank. (2010) 11(4):413–9. 10.1007/s10561-010-9226-0 21235034

[17] Directive 2004/23/EC of the European Parliament and of the Council of 31 March 2004 on setting standards of quality and safety for the donation, procurement, testing, processing, preservation, storage and distribution of human tissues and cells (2004). Available online at: https://eur-lex.europa.eu/legal-content/EN/TXT/?uri=celex%3A32004L0023 (accessed September 25, 2023).

[18] Directive 2010/45/EU of the European Parliament and of the Council of 7 July 2010 on standards of quality and safety of human organs intended for Transplantation (2010). Available online at: https://eur-lex.europa.eu/LexUriServ/LexUriServ.do?uri=OJ:L:2010:207:0014:0029:EN:PDF (accessed September 25, 2023).

[19] Real Decreto 1301/2006, de 10 de noviembre, por el que se establecen las normas de calidad y seguridad para la donación, la obtención, la evaluación, el procesamiento, la preservación, el almacenamiento y la distribución de células y tejidos humanos. (2006). BOE-A-2006-19625.

[20] Real Decreto 1723/2012, de 28 de diciembre, por el que se regulan las actividades de obtención, utilización clínica y coordinación territorial de los órganos humanos destinados al trasplante y se establecen requisitos de calidad y seguridad. BOE-A-2012-15715.

[21] Generalitat de Catalunya. Biomonitoring Register. Donation and transplantation. (2002). Available online at: https://trasplantaments.gencat.cat/ca/recursos/registres_activitat_i_seguiment/registre_de_donacio_i_trasplantament/index.html (accessed September 12, 2023).

[22] Regulation (EU) 2016/679 of the European Parliament and of the Council of 27 April 2016 on the protection of natural persons with regard to the processing of personal data and on the free movement of such data, and repealing Directive 95/46/EC (2016). Available online at: https://eur-lex.europa.eu/eli/reg/2016/679/oj (accessed September 10, 2023).

[23] Ley Orgánica 3/2018, de 5 de diciembre, de Protección de Datos Personales y garantía de los derechos digitales. BOE-A-2018-16673.

[24] Vigilance Tools Wallchart. Eustite EU-Project. Notify Library web Site. Available online at: https://www.notifylibrary.org/sites/default/files/EUSTITE%20Vigilance%20Tools%20Wallchart_0.pdf (accessed October 26, 2024).

[25] Marazuela R Domínguez-Gil B Valentín MO Martín S Perojo L Matesanz R. Recommendations on the Vigilance of Human Organs Intended for Transplantation. Deliverable 10 (Part II) (2011). Available online at: https://www.notifylibrary.org/sites/default/files/EFRETOS%20Recommendations%20on%20Organ%20Vigilance.pdf (accessed July 30, 2023).

[26] Adverse occurrence type taxonomy. (Annex 2). Notify Library-Adverse 447 occurrence type taxonomy. Available online at: https://www.notifylibrary.org/content/annex-2-notify-library-adverse-occurrence-type-taxonomy (accessed July 30, 2023).

[27] Petrisli E Carella C Navarro A Fehily D Strong DM Cardillo M. Vigilance for medical products of human origin—progress on the notify Library’s global effort to share information and learning. Transplantation. (2021) 105(9):1921–9. 10.1097/TP.0000000000003589 33449611 PMC8376274

[28] Ison MG Nalesnik MA. An update on donor-derived disease transmission in organ transplantation. Am J Transplant. (2011) 11(6):1123–30. 10.1111/j.1600-6143.2011.03493.x 21443676

[29] Project SV and surveillance. SOHO V&S Guidance for Competent Authorities: Communication and Investigation of Serious Adverse Events and Reactions associated with Human Tissues and Cells. (2013). Available at: https://www.httpnotifylibrary.org/sites/default/files/SOHO>V%26S Communication and Investigation Guidance.pdf

[30] European Directorate for the Quality of Medicines & HealthCare (EDQM). The Guide to the Quality and Safety of Tissues and Cells for Human Application. 5th ed. Starsbourg European Directorate for the Quality of Medicines & Healthcare (2022).

[31] European Group for Coordination of National Research Programmes on Organ Donation and Transplantation. Aliance-o Project. Organ donation and Transplantation policy options at EU level consultation document (June 26th, 2006) p. 1–21. Available online at: https://ec.europa.eu/health/ph_threats/human_substance/oc_organs/consultation_paper.pdf (accessed July 12, 2023).

[32] Debout A Foucher Y Trébern-Launay K Legendre C Kreis H Mourad G Each additional hour of cold ischemia time significantly increases the risk of graft failure and mortality following renal transplantation. Kidney Int. (2015) 87(2):343–49. 10.1038/ki.2014.304 25229341

[33] ABM A de la B. Rapport annuel sur le dispositif de biovigilance. (2020). Available online at: https://www.agence-biomedecine.fr/IMG/pdf/rapport_biovigilance_2020_vf1.pdf

[34] European Commission. Directorate General for Health and Food Safety. Annual Reporting of SAR and SAE for Tissues and Cells 2019. Eur Comm (2019). p. 1–27. Available online at: https://health.ec.europa.eu/system/files/2021-01/2019_sare_tc_summary_en_0.pdf (accessed August 11, 2023).

[35] Parekh M Salvalaio G Ferrari S Amoureux MC Albrecht C Fortiere D A quantitative method to evaluate the donor corneal tissue quality used in a comparative study between two hypothermic preservation media. Cell Tissue Bank. (2014) 15(4):543–54. 10.1007/s10561-014-9424-2 24567232

[36] Madzak A Hjortdal J. Outcome of human donor corneas stored for more than 4 weeks. Cornea. (2018) 37(10):1232–6. 10.1097/ICO.0000000000001676 29965863

[37] Sabater-Cruz N Otero N Dotti-Boada M Ríos J Gris O Güell JL Eye bank and theatre factors for positive microbiological culture of corneoscleral rim and cornea storage medium in the real-world. Eye (2021) 35(11):3087–93. 10.1038/s41433-020-01342-8 33469128 PMC8526809

[38] ABM A de la B. Rapport annuel sur le dispositif de biovigilance. (2021):1–57. Available at: https://www.agence-biomedecine.fr/IMG/pdf/2021_rapport_biovigilance.pdf

[39] Domini F Nazionale C. Stati Generali Della Rete Trapiantologica Italiana. (2023). p. 122–131. Available online at: https://www.trapianti.salute.gov.it/imgs/C_17_cntPubblicazioni_605_allegato.pdf (accessed June 28, 2023).

[40] Clinical Governance and the Lead Clinical Microbiologist, Organ Donation and Tissue and Transplantation Directorate (OTDT). National Heath System and Transplant. UK. Events investigated for possible donor-derived transmission of infections, malignancies and other cases of interest April 2020—March 2021 1. (2021). p. 1–9. Available online at: https://nhsbtdbe.blob.core.windows.net/umbraco-assets-corp/24453/30-june-2021-report-events-investigated-for-possible-donor-derived-transmission-of-infections-malignancies-march-2018-march-202.pdf (accessed June 19, 2023).

[41] The Australian Vigilance and Surveillance report 2022 p. 1–35. (2023). Australian Vigilance and Surveillance Framework for Organ Donation for Transplantation. Available online at: https://www.donatelife.gov.au/sites/default/files/2023-06/OTA_2022_VSEAC_Annual_Report.pdf (accessed August 20, 2023).

[42] Kaul DR Vece G Blumberg E La Hoz RM Ison MG Green M Ten years of donor-derived disease: a report of the disease transmission advisory committee. Am J Transplant. (2021) 21(2):689–702. 10.1111/ajt.1617843 32627325

[43] Centers for Disease Control (CDC). Transmission of multidrug-resistant escherichia coli through kidney transplantation—California and Texas, 2009. Morb Mortal Wkly Rep. (2010) 59(50):1642–6. 21178948

[44] Goldberg E Bishara J Lev S Singer P Cohen J. Organ transplantation from a donor colonized with a multidrug-resistant organism: a case report. Transpl Infect Dis. (2012) 14(3):296–9. 10.1111/j.1399-3062.2011.00697.x 22176504

[45] Watkins A Vedula G Horan J Dellicarpini K Pak S Daly T The deceased organ donor with an “open abdomen”: proceed with caution. Transpl Infect Dis. (2012) 14(3):311–5. 10.1111/j.1399-3062.2011.00712.x 22283979

[46] Prieto M Gómez MD Berenguer M Córdoba J Rayón JM Pastor M De novo hepatitis B after liver transplantation from hepatitis B core antibody—positive donors in an area with high prevalence of anti-HBc positivity in the donor population. Liver Transplant. (2001) 7(1):51–8. 10.1053/jlts.2001.20786 11150423

[47] Rodrigues BF Natário AS Vizinho RS Jorge CM Weigert AL Martinho A Candida species contamination of preservation fluid-outcome of renal transplantation in 6 patients. Transpl Proc. (2013) 45(6):2215–9. 10.1016/j.transproceed.2013.03.024 23953531

[48] Abanyie FA Gray EB Delli Carpini KW Yanosfky A McAuliffe I Rana M Donor-derived strongyloides stercoralis infection in solid organ transplant recipients in the United States, 2009–2013. Am J Transplant. (2015) 15(5):1369–75. 10.1111/ajt.13137 25703251 PMC4747246

[49] Peghin M Grossi PA. Donor-derived infections in solid organ transplant recipients. Curr Opin Organ Transplant. (2023) 28(5):384–90. 10.1097/MOT.0000000000001094 37555801 PMC10597443

[50] Mahíllo B Martín S Molano E Navarro A Castro P Pont T Malignancies in deceased organ donors: the Spanish experience. Transplantation. (2022) 106(9):1814–23. 10.1097/TP.0000000000004117 35421045

