# Supplementary material for: The value of organ and tissue biovigilance: a cross-sectional analysis
Source: Front Transplant. 2024 Feb 20;3:1307946. doi: 10.3389/frtra.2024.1307946 (PMC11235277; doi:10.3389/frtra.2024.1307946)
Supplement: Supplementary file 1 [file Datasheet1.pdf]

Supplementary file

Table S1. Donors, recipients, SAEs, and SARs of tissues (13 years) and organs (5 years) during the study period, *N (%).*

	Donations	Distributed/Transplants	SAEs	Total SARs/ SARs CPP	Incidence SARs ‰-1 SAR/"x" Tx
**Tissues**	**N=34 306****	**Distributed=156 763**	**N=118**	**N=36/35**	
Ocular tissue	24 656	26 372 (16.8)		(-)	
Cornea	23 737	19 501 (12.4)		20 /19	0.97- 1/ 1026
Sclera	527	2041 (1.3)		(-)	
Amniotic membrane	392	4830 (3.1)		(-)	
Musculoskeletal tissue	3521	117 171 (74.7)		10	0.08- 1 / 11717
Skin	2358	170 (0.1)		0	
Valves	2063	1950 (1.2)		2	1.02- 1 / 1/975
Arteries	1708	1657 (1.1)		2	1.2 – 1/ 825
Other (includes eye drops)	NA	9612 (6.1)		2*	NA
**Organs**	**N=2287*****	**Transplants=5569**	**N=72**	**N=53/49**	
Kidney	3072	3822 (68.6)		33(30)	7.8 – 1 / 127.4
Liver	966	894 (16.1)		11(10)	11.2 – 1/ 89.4
Lung	829	459 (8.2)		5	10.9- 1/ 91.8
Heart	256	292 (5.2)		3	10.3 – 1/ 97.3
Pancreas	127	102 (1.8)		1	9.8 – 1/ 102

*Serum eye drops; **Deceased donors; *** Living and deceased donors; (-), no data; NA, not available; CPP. Certain, probable, possible; "x", number; Tx, transplants

Table S2. Detailed characteristics of serious adverse events related to tissue donation involving a risk from the donor (A) or tissue (B) and actions implemented following their notification.

	n	Criteria[a]	Stage	Cause	I[bc]	Investigations and proposed actions by BV advisory committee
**A) Donor-derived risk**						
Blood Tests	24					
Viral						
Hepatitis B virus (DNA)	6	1, 2	Tests	DD	6	Discard tissues. Follow-up of organ recipients (2K) and, if required, prophylactic Tt: lamivudine. Implement NAT
Hepatitis C virus (RNA)	2	1	Tests	DD /Material	6	Initial result positive. Alert Tx centers 2CT, L, and 2K. Confirmatory RNA negative (non-reliable initial kit)
Human immunodeficiency virus (NAT)	1	1	Tests	DD	6	Initial positive HIV NAT testing. Even confirmed false positive, MSK and 2CT discarded
Antibodies to hepatitis C virus	1	1, 2	Tests	DD	6	Discard tissues. Follow-up of organ recipients (L, 2K).
Antibodies to hepatitis B virus (HBc positive, HBs negative)	1	1, 2	Tests	DD	6	Discard tissues (2CT).
Bacterial						
Syphilis (Treponema pallidum Ab)	9	1, 2	Tests	DD	6	Before donation routine RPR test negative. Discard tissues. Follow-up of 2CT, MSK and organ (6K, 2L) recipients.
Parasite						
Antibodies to *Toxoplasma gondii* (IgM)	3	1, 2	Tests	DD	4	Discard 2CT. Follow Lu, L, Pc, and 4K recipients.
Antibodies to *Trypanosoma cruzi*	1	1, 2	Tests	DD	4	Discard tissues. Follow-up of 2CT recipients. If required, prophylactic Tt of organ (2K) and MSK (F) recipients with benznidazole, trimethoprim/sulfamethoxazole.
Blood cultures	14					
Clostridium sp.	5	1, 2	Tests	DD	6	Discard tissues. Follow-up of 10CT recipients. If Tt required: prophylaxis with clindamycin.
Enterococcus faecalis	2	1	Tests	DD	6	Discard tissues. Prophylaxis of 2CT recipients with vancomycin.
Candida spp.	2	1, 2	Tests	DD	6	Discard tissues. Follow-up and, if required, Tt of organ (2K) and tissue (2CT) recipients.

Enterobacter cloacae	1	1, 2	Tests	DD	6	Discard tissues. Follow-up of organ (2K) recipients.
Staphylococcus aureus	1	1, 2	Tests	DD	6	Follow-up of organ (2K) and tissue (2CT) recipients.
Granulicatella adiacens	1	1, 2	Tests	DD	6	Prophylaxis 2CT recipients with linezolid.
Staphylococci lugdunensis	1	1	Tests	DD	6	Prophylaxis 2CT recipients with ciprofloxacin or levofloxacin.
Bacillus cereus	1	1	Tests	DD	6	Discard tissues and CT. Follow-up of MSK (2 Fz patella) recipients.
Bronchial aspirate cultures	2					
Mucor spp.	1	1, 2	Tests	DD	3	Discard tissues. Follow-up of organ (Lu, H, L, and 2K) recipients.
Mycobacterium tuberculosis	1	1	Tests	DD	6	Prophylactic Tt of 2CT recipients with isoniazid/rifampicin.
Malignancies	15					
Prostate adenocarcinoma	12	1, 2	Tests	DD	6	Tx 22CT recipients. Discard tissues. Follow-up of organ (Lu, 7L, 14K) recipients for ≥2 years.
Nodule not assessed (lung)	1	2	Tests	DD	6	Discard tissues (HV). Follow-up of organ (L, 2K) recipients.
Donor metastasis	1	1, 2	Tests	DD	4	Tx of 2CT recipients. Discard tissues (HV). Follow-up of organ (L, 2K) recipients.
Unexpected hepatocarcinoma	1	2	Tests	DD	4	Discard tissues (MSK, HV).
Donor medical contraindications	6					
Tuberculosis	2	1, 2	DE	DD	6	Discard tissues. Prophylactic Tt of organ (L, 2K) recipients with isoniazid, rifampin/rifabutin and ethambutol. Follow-up of 4CT recipients.
Monoclonal gammopathy	1	1	DE	DD	3	Discard tissues (skin and MSK tissue). Follow-up of 1CT recipient.
Lymphocyte meningoencephalitis (identified at autopsy)	1	2	DE	DD	4	Follow-up of organ (L, 2K, 2CT) recipients.
Alzheimer's disease (identified at autopsy, 4 months post-donation)	1	1, 2	DE	DD	4	Discard tissues (MSK, HV, skin). Follow-up of organ (2K) and 2CT recipients.
Infectious myocarditis (identified at autopsy)	1	1, 2	DE	DD	4	Follow-up of organ (L, 2K) and 2CT recipients.

SARs in organ recipients	4					
Liver cholangiocarcinoma identified in L recipient (explanted 4 years after Tx)	1	2	SAR	DD	4	No action taken.
Millar tuberculosis in K recipient (identified 4 months after Tx)	1	1, 2	SAR	DD	4	Discard tissues (MSK, Skin). Prophylactic Tt of organ (L, 2K) recipients. Follow-up of MSK, ART and 2CT recipients.
Creutzfeldt-Jakob disease in L recipient (identified 4 years after Tx)	1	1	SAR	DD	8	Follow-up of HV recipient.
Adenocarcinoma (identified in 2K recipients 2.5 years after Tx)	1	2	SAR	DD	4	Follow-up of 2CT, 28MSK (Fz), 13MSK (Ly), and 1H recipients. NT at 5y follow-up.
**B) Tissue-derived risk**						
Tissue culture at recovery	7					
Candida spp. (2HV, 1ART)	3	1	Recovery	TQ	6	Discard tissues. Follow-up of 2CT and 3MSK tissue recipients.
Clostridium spp. (1 ART, 2MSK, CT)	3	2	Recovery	TQ	6	Discard tissues (2MSK, HV, ART). Prophylaxis of organ (Lu, H, LK, PcK, 4K) and 5CT recipients with clindamycin.
Staphylococcus aureus (2CT)	1	1	Recovery	TQ	6	Prophylaxis in 2CT recipients with erythromycin.
Processing culture	5					
Clostridium spp. (skin, pericardium, and MSK tissue)	3	1, 2	Processing	TQ	6	Discard tissues (2MSK, ART, HV, skin). Follow-up of organ (2K) recipients and 2CT. Prophylaxis in 4CT recipients with clindamycin.
Aspergillus spp. (MSK and ART tissue)	1	1, 2	Processing	TQ	6	Discard tissues (MSK, ART). Follow-up of 2CT, L, 2K and H recipients.
Cupriavidus metallidurans (MSK tissue processing solution). Contaminated	1	1, 2, 5	Processing	Material	8	Follow-up of 213MSK tissue recipients. Change in procedures (sterility test processing solution, 40 donors immobilize, alert other TEs if same solution used).
culture at the TE	7					
GPB (CT)	1	1, 2	Processing	TQ	6	Follow-up of 1K recipient. Prophylactic Tt of 1CT recipient with azithromycin.

Granulicatella adiacens (CCT)	1	1	Processing	TQ	4	Prophylactic Tt of 1CT recipient with linezolid.
Klebsiella pneumoniae (scleral tissue from FCT)	1	2	Processing	TQ	6	Prophylactic Tt (2CT) with intra-camera cefuroxime at the end of surgery and postoperative Tt with moxifloxacin. Follow-up of organ (2K) recipients.
Serratia marcescens (scleral tissue from FCT)	1	2	Processing	TQ	6	Follow-up of organ (2K) and tissue (2CT) recipients.
Candida spp. (CCT)	1	1	Processing	TQ	6	Follow-up of 1CT recipient.
Rhodotorula spp. (CCT)	1	1	Processing	TQ	6	No action taken.
Burkholderia cepacia (CCT)	1	1, 2	Processing	SF	4	Prophylactic Tt of 4CT recipients with cotrimoxazole.
Culture at the TC	22					
Candida spp. (CT and bronchoscopy culture)	5	1	Culture TC	TQ	6	Change procedures (avoid cross-contamination at recovery). Prophylactic Tt of tissue (9CT) recipients with fluconazole. Follow-up of organ (1K) recipient.
Clostridium spp. (HV, MSK tissue)	2	1	Culture TC	TQ	6	Prophylaxis of tissue (4CT, 1MSK) recipients with clindamycin.
Coagulase-negative *Staphylococcus* (AM, CT)	2	1	Culture TC	TQ	4	Follow-up of organ (2K, 1AM, 2CT) recipients. Change in procedures (sample testing).
Escherichia coli (MSK tissue, CT)	2	1	Culture TC	TQ	6	Follow-up of tissue (4CT) recipients and prophylactic Tt if required with cefixime. If symptoms, ertapenem.
Exophiala spp. (CT)	2	1, 2	Culture TC	TQ	6	Follow-up of tissue (4CT) recipients and, if required, prophylactic Tt with voriconazole/isavuconazole/posaconazole.
Multi-drug resistant *Staphylococcus epidermidis* (CT)	1	1, 2	Culture TC	TQ	6	Prophylactic Tt of tissue (2CT) recipients with linezolid.
Streptococcus pyogenes (MSK tissue)	1	1, 2	Culture TC	TQ	6	Prophylactic Tt of tissue (8MSK) recipients with amoxicillin/cephalosporin. Follow-up of tissue (2ART) recipients.
Chryseobacterium spp. (CCT)	1	1, 2	Culture TC	TQ	4	Prophylactic Tt of tissue (2CT) recipients with ciprofloxacin and topical moxifloxacin.
Pseudomonas and MRSA (MSK tissue)	1	1, 2	Culture TC	TQ	6	Follow-up of tissue (4MSK, 2CT) recipients.

Proteus mirabilis and *Escherichia coli* (CT medium)	1	1, 2	Culture TC	TQ	4	Follow-up of tissue (1CT) recipient.
Bacillus cereus (MSK tissue)	1	1, 2	Culture TC	TQ	6	Follow-up of tissue (2CT, MSK, ART) recipients.
Acinetobacter lwoffii (CT)	1	1, 2	Culture TC	TQ	6	Follow-up of tissue (2CT) recipients.
Aureobasidium pullulans (CT)	1	1, 2	Culture TC	TQ	6	Follow-up of tissue (2CT) recipients.
Microorganism NA (color change of the preservation solution)	1	1	Culture TC	HE	4	Change in procedure (review CT validation).
Other	12					
Tissue cross-over (left vs. right and proximal vs. distal)	2	3	Issue	SF	4	Change in procedures (staff training).
Sealing defect (cranial flap)	2	1, 4	Tissue validation	SF	4	Follow-up of 1 cranial flap recipient. Review procedures (sealing).
Corneal defect at TC. Tx cancelled	1	1	Tissue validation	HE	4	Review procedures (quality validation).
Loss of tissues (55DBM vials)	1	5	Transport	SF	4	Change in procedures (add checklist verification).
Tissue transplanted after the expiration date (MSK tissue)	1	3	Issue	SF	4	Follow-up of tissue (MSK) recipient.
Tendon rupture during Tx	1	4	Tissue validation	TQ	4	Review procedure (tendon validation).
HV not found during distribution	1	4	Issue	SF	6	Review procedure (add checklist once tissue matched).
DBM without osteoinductivity	1	1, 5	Tissue validation	SF	4	Review procedure (validation demineralization).
Fz CT arrives at TC	1	4	Transport	HE	4	Review procedure (staff training).
Damaged 28CCT due to an increase in the cell incubator temperature	1	5	Storage	EF	6	Discard equipment (design error in the control panel).

[a] Criteria for reporting SAEs as described in the SoHO V&S guidelines[2]: 1, inappropriate tissues/cells have been distributed for clinical use; 2, the event could have possible implications for other patients or donors; 3, the event resulted in a mix-up of tissues/cells; 4, the event resulted in loss of any irreplaceable autologous tissues or cells or any highly matched (i.e. recipient specific) allogeneic tissues or cell; 5, the event resulted in the loss of a significant quantity of unmatched allogeneic tissues/cells. [b] Potential impact calculated by multiplying likelihood of recurrence and the highest score of individual, system and distribution consequences (Eustite V&S tools v2.1c). Impact classification: minor (0-3), moderate (4-6), moderate-high (8-9), or extreme (10-20) impact. [c] https://www.notifylibrary.org/sites/default/files/EUSTITE%20Vigilance%20Tools%20Wallchart_0.pdf

AM, amniotic membrane; AT, arterial tissue; GPB, Gram-positive bacillus; CT, corneal tissue; CCT, cultured corneal tissue; CV, cardiovascular; DBM, demineralized bone matrix; DE, donor evaluation; F, fresh; Fz, frozen; H, heart; HBc, hepatitis B core antigen; HBs, hepatitis B surface antigen; HV, heart valves; I, impact; K, kidney; L, liver;; Lu, lungs; Ly, lyophilized; MRSA, methicillin-resistant *Staphylococcus aureus*; MSK, musculoskeletal; NA, not available; NAT, nucleic acid testing; NT, no transmission; SAR, severe adverse reaction; DD, Donor disease without transmission,; TQ, tissue quality and/or safety; TC, transplant center; TE, tissue establishment; Tt, treatment; Tx, transplant.

Table S3. Description of serious adverse reactions related to tissue transplantation N=36.

Notify library taxonomy: Harm to a recipient (Level #1) - adverse occurrence taxonomy

Level #1	Level #2	Level #3	Level #4	Type of tissue	Detection time (days)	Imputability[d]	I[a]	Re-transplant
Harm to a recipient (36)	Infection (22)	Fungal (11)	*Candida* spp.*(9)*<br>ND (2)	CT (6)<br>CCT (4)<br>FCT (1)	1-90	Probable (6)<br>Possible (4)<br>Unlikely (1)	4-6	Yes (5)<br>No (1)<br>NA (3)
		Fungal and bacterial (1)	*Fusarium* spp +<br>*Pseudomonas* spp.	CT (1)	<30	Probable	9	No
		Bacterial (8)	*Bacillus cereus*<br>*Bacteroides stercoris*<br>Bacterial, ND *Clostridium* spp.<br>*Enterobacter cloacae (2)*<br>*Staphylococcus epidermidis*<br>*Proteus mirabilis* +<br>*Staphylococcus lugdunensis*	FCT<br>ICW (Fz)[c] (2)<br>Proximal femur (Fz)<br>Long peroneal tendon (Fz)<br>ICW (Fz)<br>Autologous serum (eye drops)<br>DBM (Ly)[b]	<30	Certain (1)<br>Probable (4)<br>Possible (3)	4-6	NA
		Infection, ND (2)		FCT<br>Autologous serum (eye drops)	<30	Certain Probable	4-6	No<br>NA
	Miscellaneous (14)	Undue exposure to risk Transplant cancelled (3)	Cornea (poor quality)<br>Osteochondral (Fz versus F graft)<br>Achilles tendon (without calcaneus)	FCT<br>Osteochondral (F)<br>Achilles tendon (Fz)	0	Certain-Process related (2)<br>Probable	4	No<br>NA (2)

		Graft failure (7)	Descemet membrane detachment<br>Edema<br>Early tendon rupture<br>Late tendon rupture	FCT(2)<br>CCT (3)<br>Patellar tendon (Fz)<br>Long peroneal tendon (Fz)	0-300	Probable (5)<br>Possible (2)	4-6	Yes (4)<br>NA (3)
		Cardiovascular complications (4)	Thrombosis<br>Rupture (3)	Femoral artery<br>Iliac artery<br>Pulmonary heart valve<br>Aortic heart valve	0-40	Certain (2)<br>Probable (1)<br>Possible (1)	6-8	No
	[a] Impact minor: 0-3; moderate: 4-6; moderate-high: 8-9; extreme: 10-20; calculated by multiplying likelihood of recurrence (score 1-5) and the highest score of individual, system and distribution consequences (score 0-4) from Eustite V&S tools (https://www.notifylibrary.org/sites/default/files/EUSTITE%20Vigilance%20Tools%20Wallchart_0.pdf) [b] Facial cellulitis after tooth extraction. [c] Surgical wound exudate. [d] Imputability: SOHO V&S Guidance for Competent Authorities: Communication and Investigation of Serious Adverse Events and Reactions associated with Human Tissues and Cells source (https://www.notifylibrary.org/sites/default/files/SOHO%20V%26S%20Communication%20and%20Investigation%20Guidance.pdf); CCT, cultured corneal tissue; CT, corneal tissue; F, fresh; FCT, fresh/hypothermic corneal tissue; Fz, frozen; I, impact; NA, not assessable; Tx, transplant.							

Table S4. Serious adverse events related to organ donation according to the type of organ donor. *N (%).*

DBD (N=1031)	DCD (N=665)	Living (N=663)	*P*
41 (3.9)	30 (4.5)	1 (0.2)	<0.0001

DBD, donation after brain death; DCD, donation after circulatory death.

Table S5. Detailed characteristics of serious adverse events related to organ donation involving a risk from the donor (A) or organ (B) and actions implemented after their notification

	n	Criteria[a]	Stage	Cause	I[b]	Investigations and proposed actions by BV advisory committee
**A) Donor-derived risk**						
Blood tests	19					
Viral	8					
Hepatitis B virus, HBc positive, HBs negative	1	3	Blood testing	DD	6	Follow-up and monitoring of serology in organ (H, 2K) recipients.
Hepatitis B, HBsAg positive	1	3	Blood testing	HE	6	First result positive. Only 2 Lu accepted. Confirmatory test: negative. It was a transcription error (human error) and for this a SAE was opened to review procedures and train staff.
Hepatitis B virus, DNA positive HBcore positive	1	3	Blood testing	EF	6	Initial DNA positive. Confirmed negative. Review organ (BiLu, L, 1K, 1KPc) vaccination and serology monitoring. Discard tissues (C, MSK, HV, skin, ART). If required, prophylactic Tt with lamivudine.
Hepatitis B virus DNA positive	1	3	Blood testing	DD	6	Review organ (2K) vaccination and serology monitoring. Discard tissues (CT, MSK, HV, skin, ART). If required, prophylactic Tt with lamivudine; if seroconversion, Tt with entecavir.
Antibodies to hepatitis C virus	1	2	Blood testing	HE	3	Donation accepted as HCV Ab negative (transcription error). After Tx repeated and confirmed serology positive. Prophylactic Tt of Lu recipient: velpatasvir, sofosbuvir and glecaprevir, Pibrentasvir for 2K and serology monitoring.
Human immunodeficiency virus (NAT)	1	2	Blood testing	EF	4	Initial result positive. Confirmatory test negative (SAE investigation detected non-reliable test kit at the transplant centre)
Antibodies to cytomegalovirus	1	1	Blood testing	DD	3	Repeat and confirm serology (initial false negative), review organ (2K) serology, PCR monitoring and prophylactic Tt with valganciclovir (paediatric recipients).
Antibodies to Human T-lymphotropic virus types 1 and 2 (performed at TE)	1	1	Blood testing	DD	4	Discard of tissues (CT, MSK). NT to organ (K) recipient due to Tt with antiretroviral therapy.

COVID-19 (PCR)	1	1	Blood testing	SF	6	Review procedure. Add checklist before Lu transplantation.
Bacterial	4					
Syphilis (Treponema pallidum Ab)	4	3	Blood testing	DD	6	RPR negative rutine pre-transplant donor screening. Prophylactic Tt of organ (7K, 2L) recipients with penicillin G benzathine. Discard tissues (4CT, 4MSK, 2HV, 3skin).
Parasite	7					
Strongyloides stercoralis	1	3	Blood testing	DD	6	Prophylactic Tt of organ (PcK) recipient with ivermectin. NT.
Strongyloides stercoralis, Plasmodium falciparum	1	3	Blood testing	DD	6	Initially positive both parasites. Lu, L, 2K recipients start prophylactic Tt with artemether/lumefantrine and ivermectin. Confirmatory test reveals plasmodium negative so Tt cancelled. SAE opened to validate Plamodium Real Time PCR Detection Kit Discard tissues (C, MSK).
Strongyloides, Schistosoma	1	3	Blood testing	DD	6	Prophylactic Tt organ (Lu, L, 2xK) recipients with ivermectin. NT. Discard tissues (2C, MSK, HV, ART, skin).
Schistosomiasis/	1	3	Blood testing	DD	6	No Tt for schistosomiasis (BiLu, L, 2K). Discard tissues (2CT, MSK, HV, skin).
Antibodies *Toxoplasma gondii* (IgM)	1	3	Blood testing	DD	4	Prophylactic Tt of MSK recipients with trimethoprim/sulfamethoxazole. Organs already covered. Follow-up of organ (BiLu, L, 2K) recipients.
Antibodies *Leishmania* spp.	1	3	Blood testing	DD	2	No actions taken in organ (2K) recipients.
Blood cultures	1					
Enterobacter cloacae	1	3	Culture	DD	6	Prophylactic Tt with carbapenems in organ (BiLu, 2K) recipients. Discard tissues (C, HV, MSK, Skin ART).
Incomplete donor characterization	6					
Missing samples for cross-matching	4	2	Donor charact.	SF	4	Review procedures and staff training (5K). 1K was not transplanted because it was for a hyperimmunized recipient.
Missing samples for anti-EBV	2	2	Donor charact.	SF	4	Review procedures and staff training. Follow-up of organ (3K) recipients.
HLA	1					

Error in HLA donor result interpretation	1	2	Donor charact.	HE	3	Review procedures and staff training. Creation of a new decision algorithm when new techniques implemented. 1K recipient was hyperimmunized and could not be transplanted.
Bronchoscopy culture	4					
Mycobacterium tuberculosis	1	3	Culture	DD	6	Prophylactic Tt recommended for organ (2Lu, LK, K) recipients with isoniazid, rifampin, rifabutin and ethambutol.
Citrobacter freundii	1	3	Culture	DD	6	Prophylactic Tt of organ (BiLu, H, L, 2K) recipients with ertapenem/ciprofloxacin in
Influenza A virus	1	3	Culture	DD	6	Prophylactic Tt in organ (Lu) recipient with oseltamivir.
Bacillus cereus	1	3	Culture	DD	6	Prophylactic Tt with meropenem in organ (H, L, 2K) recipients. Discard tissues (2CT, MSK, Skin ART).
Pathology	23					
Mycobacterium avium (ganglion)	1	3	Pathology	DD	6	Prophylactic Tt of organ (Lu, LK, K, NT at 7y) recipients with azithromycin/ethambutol/rifampicin.
Breast adenocarcinoma[c]	1	4	Autopsy	DD	6	Discard (MSK, HV) tissues except for CT. Follow-up of organ (L, 2K, NT at 5y) recipients.
Gastrointestinal stromal tumor	1	4	Autopsy	HE	4	Review procedure (establish early alert). Follow-up of organ recipients 2K (NT at 5y).
Colon adenocarcinoma (living donor)	1	4	Living donor follow-up	DD	6	Follow-up of 1K recipient (NT at 5y+5m).
Enteral adenocarcinoma[c]	1	4	Autopsy	DD	8	Liver-kidney explant and retransplantation in 3 weeks (LK,1K, NT at 7y).
Severe dysplasia (esophagus)	1	4	Autopsy	DD	4	Review procedure (establish early alert). Follow-up of organ (Lu, L, 2K, NT at 6y) recipients.
Neuroendocrine pancreatic tumor (initially benign)[c,d]	2	4	Pathology	DD	4	Follow-up of organ (Lu, 2K, L, NT at 5y) recipients. One case (Lu recipient) died due to other causes.
Hepatocellular adenocarcinoma[c,e]	1	4	Autopsy	DD	6	Follow-up of 2K recipients (NT at 5 years). Discard tissues (MSK, HV), except for CT.

Thyroid carcinoma[e]	1	2	Donor charact.	HE	8	Follow-up of organ recipients (Lu, H, 2K, NT at 2y 8m).
Hodgkin's lymphoma[e,f]	1	4	Pathology	SF	6	Follow-up of organ recipients (Lu, L, Pc-K, K, NT at 2y+7m). Monitoring of organ recipient liquid biopsies.
Prostatic adenocarcinoma	10	4	Pathology	DD	6	Follow-up of organ recipients (19K, 3L, 4Lu, NT from 6y to 2y+4m) and discard tissues (10MSK, 4HV,5SK, ART), except for (10CT).
Prostatic intraepithelial neoplasia	2	4	Pathology	DD	6	Discard tissues (2MSK, HV, 2SK, ART). Follow-up of organ recipients (3K, 1L, 1Lu, NT at 3y) except for 2CT.
**B) Organ-derived risk**						
Malignancy	8					
Renal cell carcinoma	4	4	Procurement	OQ	6	Discard tissues (MSK) except for CT. Follow-up of organ recipients (2K, 1L, NT from 2-6y).
Papillary renal carcinoma	2	4	Procurement	OQ	6	Discard tissues (2MSK, HV) except for CT. Follow-up of organ recipients (3Lu, 2L, 1H, 2K, NT at 4y).
Cystic renal tumor	1	4	Pathology	OQ	4	Discard tissues (HV) except for CT. Follow-up of organ recipients (L, 1K, NT at 2y).
Hepatocarcinoma	1	4	Pathology	OQ	4	Discard tissues (MSK) except for CT. Follow-up of organ recipients (2K, NT at 5y)
Lesion or alteration of vascular function	4					
Sectioned artery	2	1	Procurement	HE	6	Artery repair, with good functionality (2K).
Kidney injury due to an inappropriate biopsy	1	1	Procurement	HE	4	Cancellation of Tx (1K).
Necrotizing glomerulonephritis	1	1	Perfusion	HE	6	Cancellation of Tx (1K).
Perfusion	1					
ESBL-producing *Klebsiella pneumoniae*	1	1	Preservation solution	DD	6	Treatment of organ (K) recipient with ceftazidime/avibactam for 21 days.
Preservation or packaging	5					
Fz kidney	2	2	Preservation	SF	4	Review procedure for organ preservation and packaging. Staff training.(2K).
Kidney at room temperature	1	2	Preservation	SF	4	Review procedure for organ preservation and packaging. Staff training (1K).
Fz lung and packaging deficiency	1	2	Preservation	SF	4	Review procedure for organ preservation and packaging. Staff training (Lu).

Lung packaging deficiencies (Fz and not sterile)	1	2	Preservation	SF	4	Review procedure for organ preservation and packaging. Staff training (Lu).

[a] Criteria for reporting organ SAEs as described in the EFRETOS project: 1, deviations from operating procedures or other adverse event during the chain from donation to transplantation that might lead to a SAR; 2, deviations in operating procedures or steps during the chain from donation to transplantation, with a potential high impact on the health of the patient and easy to be prevented; 3, infection or positive serological status discovered in an organ donor when ≥1 organ has been transplanted; 4, malignant tumor discovered in an organ donor when ≥1 organ has been transplanted; 5, discovery of any other potentially transmissible disease in an organ donor when ≥1 organ has been transplanted; 6, other. [b] Potential impact calculated by multiplying likelihood of recurrence and the highest score of individual, system and distribution consequences (Eustite V&S tools v2.1c). Impact classification: minor (0-3), moderate (4-6), moderate-high (8-9), or extreme (10-20) impact. chttps://www.notifylibrary.org/sites/default/files/EUSTITE%20Vigilance%20Tools%20Wallchart_0.pdf
[c] Unacceptable risk according to the guide to the quality and safety of organs for transplantation of the Council of Europe. [d] Pancreas for research. [e] Analysed due to a history of cirrhosis. [f] Previously identified as thymoma.

CA, competent authority; COVID-19, coronavirus disease 2019; Charact., characterization; CT, corneal tissue; DC, donation center; EF, equipment failure; ESBL, extended spectrum beta-lactamase; Fz, frozen; HE, human error; PCR, polymerase chain reaction; I, impact; LR, likelihood of recurrence; NAT, nucleic acid testing; OQ, organ quality; DD, Donor disease without transmission; SF, system failure; SOP, standard operating procedures; TC, transplant center; TE, tissue establishment; Tt, treatment; Tx, transplant; y, years

Table S6. Description of organ serious adverse reactions

Notify library taxonomy: Harm to a recipient (Level #1) - adverse occurrence taxonomy				**Organ**	**Detection Time**	**Imputability[b]**	**Impact[c]**
Level #1	*Level #2*	*Level #3*	*Level #4*				
Harm to a recipient	Infection transmission (17)	Bacterial (5)	*Escherichia coli* (2)<br>ESBL-producing *Klebsiella pneumoniae*<br>*Pseudomonas aeruginosa*<br>*Enterococcus faecium*	Kidney (2)<br>Liver (3)	1-14	Certain (3)<br>Probable (2)	4-6
		Viral (4)	Hepatitis C virus<br>Herpes simplex virus<br>Hepatitis B virus (2)	Kidney<br>Liver x3	30-850	Excluded (1)<br>Probable (3)	4-8
		Parasitic (4)	*Strongyloides stercoralis*<br>*Plasmodium falciparum*	Kidney x3<br>Heart	15-120	Certain (4)	6-12
		Fungal (4)	*Mucor* spp.<br>*Aspergillus* spp.<br>*Histoplasma* sp.<br>*Candida tropicalid*[a]	Liver<br>Lung<br>Heart<br>Pancreas-kidney	4-1700	Excluded (1)<br>Certain (1)<br>Possible (2)	4-6
	Malignancy (13)	Blood and lymphoid (2)<br>Carcinoma of unknown primary site (1)<br>Gastrointestinal (3)<br>Kidney and urinary tract (3)<br>Liver, gallbladder and bile ducts (1)<br>Lung and lower respiratory tract (1)<br>Soft tissue/sarcoma (2)	Diffuse B-cell lymphoma<br>Adenocarcinoma unkown origin<br>Small bowel adenocarcinoma<br>Cholangiocarcinoma<br>Renal cell carcinoma<br>Space-occupying lesions<br>Small cell carcinoma<br>Kaposi's sarcoma	Kidney (8)<br>Lung (2)<br>Liver (3)	17-1460	Excluded (2)<br>Certain (5)<br>Probable (3)<br>Possible (3)	4-8

	Miscellaneous complication (21)	Undue exposure to risk Transplant cancelled (8)	Poor perfusion and macroscopic appearance (unsuitable).<br>Poor perfusion due to air entering into the circuit.<br>Right kidney with 3 unpatched arteries.<br>Left kidney with multiple cysts.<br>Capsule and vein defects<br>Wrong ABO recipient in waiting list<br>Incorrectly diagnosed cholangiocarcinoma; identified next day as adenoma (lost opportunity to transplant)<br>Inappropriate clinical application	Kidney (6)<br>Liver (1)<br>Lung (1)	-	Certain-Process (8)	4-8
		Delayed graft function (4)	Vein injury. Inappropriate perfusion and packaging<br>Bleeding due to decapsulation and overly large biopsy (3)	Kidney (4)	-	Certain-Process x4	6
		Other: explant (5)	Incompatible ABO. Explanted in 3h<br>Thrombosis<br>Unappropriated preservation (thrombosis)<br>Lung and lower respiratory tract malignancy[d]	Kidney (5)	1-26	Certain-Process (3)<br>Certain (2)	4-6

		Surgical site complications (4)	Artery dissection, unable to be repaired<br>Cysts occupying half of the surface<br>Complex venous access and inappropriate perfusion<br>Renal vein artery bleeding in the sinus, unable to be repaired	Kidney (4)	-	Certain-Process (4)	4
	Immunological complications (2)	Detrimental immunization	ABO immunization<br>HLA immunisation	Lung<br>Kidney	1-2	Certain-process (2)	6

[a]Presenting with acute pancreatitis and requiring an explant. [b] Imputability. Table 1: DTAC classification of donor-derived disease transmissions Disease Ison MG, Nalesnik MA. An update on donor-derived disease transmission in organ transplantation. Am J Transplant. 2011;11(6):1123–30. [c] Impact. minor: 0-3; moderate:4-6; moderate-high:8-9; extreme: 10-20; calculated by multiplying likelihood of recurrence (score 1-5) and the highest score of individual, system and distribution consequences (score 0-4) from Eustite V&S tools (https://www.notifylibrary.org/sites/default/files/EUSTITE%20Vigilance%20Tools%20Wallchart_0.pdf). [d] Two kidneys explanted one month after transplantation due to a donor autopsy revealing a lung malignancy. Both recipients were re-transplanted three and four years after explant and none had malignancy transmission after 7 years of close monitoring.ESBL, extended-spectrum-β-lactamase; Fz, frozen; I, impact; HLA, human leucocyte antigen; SAR,serious adverse reaction; Tx, transplant
